# Inactivation efficacy of atmospheric air plasma and airborne acoustic ultrasound against bacterial biofilms

**DOI:** 10.1038/s41598-021-81977-z

**Published:** 2021-01-27

**Authors:** Apurva D. Patange, Jeremy C. Simpson, James F. Curtin, Catherine M. Burgess, P. J. Cullen, Brijesh K. Tiwari

**Affiliations:** 1 Food Chemistry and Technology Department, Teagasc Food Research Centre, Ashtown, Dublin, Ireland; 2 School of Biology and Environmental Science, University College Dublin, Dublin, Ireland; 3 School of Food Science and Environmental Health, Technological University Dublin, Dublin, Ireland; 4 Food Safety Department, Teagasc Food Research Centre, Ashtown, Dublin, Ireland; 5 School of Chemical and Biomolecular Engineering, The University of Sydney, Sydney, Australia

**Keywords:** Microbiology, Bacteria, Biofilms

## Abstract

Biofilms are complex microbial communities that present serious contamination risks to our environment and health. In this study, atmospheric air plasma and airborne acoustic ultrasound technology were applied to inactivate *Escherichia coli* and *Listeria innocua* biofilms. Both technologies were efficient in controlling, or completely inactivating, the target bacterial biofilms. Viability and metabolic assays, along with microscopy analysis, revealed that atmospheric air plasma and airborne acoustic ultrasound damaged both the bacterial biofilm cells and its structural integrity. Scanning electron microscopy images highlighted the disruption of the biofilms and pore formation in bacterial cells exposed to both the plasma and acoustic treatments. Elevated reactive oxygen and nitrogen species in bacterial cells treated with atmospheric air plasma, demonstrated their primary role in the observed bacterial inactivation process. Our findings provide potential antimicrobial strategies to combat bacterial biofilms in the food and healthcare sectors.

## Introduction

Biofilms consist of a diverse variety of bacteria embedded together in a gel-like matrix of extracellular polymeric substances (EPS) and nucleic acids. They aggregate and grow in micro-colonies by attaching to abiotic or biotic surfaces or interfaces, causing a wide variety of difficulties for industrial and clinical settings. Active dispersal mechanisms of biofilms result in the colonisation of new areas, as well as acting as an important source of cross-contamination. Biofilms are responsible for several chronic infections, food spoilage, and equipment damage, resulting in considerable economic and public health problems^1–3^. Biofilms are highly organised structures; their self-produced EPS serves as a multi-layer physical barrier for the embedded bacteria against environmental and antimicrobial stresses^4^. Conventional methods of decontamination are often ineffective or inadequate for biofilms due to their complex composition and structure^5,6^. The persistence of infections and resistance to conventional treatments enabled by biofilm formation are major causes of treatment failure. Protected cells serve as a reservoir for continuous contamination and provide an environment where antimicrobial-injured cells can repair cellular damage and re-grow. In an effort to find an efficient method to control biofilm, several technology-based methods have emerged as potential alternatives to traditional treatment methods.

The antimicrobial nature of cold plasma technology has been well demonstrated in several recent studies^7–9^. Different types of plasma reactors have been used for disinfection and decontamination of microorganisms depending, on the substrate treated and their applications in the areas of biological decontamination. Plasma effects on bacterial cells are mediated by biological activators such as charged particles, electrons and ions, electric field, UV radiation and reactive oxygen and nitrogen species (RONS) such as hydrogen peroxide, singlet oxygen molecules, nitrate and nitrite^10^. Adverse influences of atmospheric air plasma (AAP) on bacteria include cellular membrane damage, alteration in structure, biological and genetic responses. Due to the numerous inactivation mechanisms, AAP has proved to be a promising treatment technology against most spoilage and pathogenic bacteria.

Ultrasound has demonstrated successful application in the food industry for drying, extraction defoaming and decontamination^11,12^ as well as in clinical settings for chronic wound care and healing^13,14^. The ultrasound produces pressure sound waves with frequencies of 20 kHz or more; with a threshold limit beyond human hearing. The bactericidal effect of ultrasound is mainly due to mechanical and chemical energy including shock waves, shear stress, pressure, agitation, vibration and cavitation^15^. Stable cavitation and pressure create multidirectional acoustic micro-jets that puncture the proximal bacterial cell membrane and damage microorganisms^16^. Several studies have examined various contact-type ultrasound systems. However, recent technology advances have shown the benefits of using non-contact airborne acoustic ultrasound for various food and non-food applications^11^. Acoustic airborne ultrasound employs non-contact transducers transmitting ultrasonic waves to treat products, using air as the coupling medium. Moreover, the use of airborne acoustics ultrasound does not restrict treatment to liquids or solid immersed in liquids but can be applied to products where there are no liquids by using tuned transducers. Despite the growing literature, information on the efficacy of non-contact acoustic airborne ultrasound against microbes, and specifically against bacterial biofilms, is limited. This study presents a comprehensive investigation of two air-based non-thermal technologies namely atmospheric air plasma (AAP) and airborne acoustic ultrasound (AAU) against early and mature bacterial biofilms grown on inanimate surfaces.

## Results and discussion

### Effect of plasma frequency variation on physiochemical properties

The pH values for distilled water decreased from 6.5 to 3.0 or 2.74 or 2.70, depending on the system frequency. There was no significant difference observed between the pH measured at different plasma treatment frequencies. The low pH values suggest increased RONS concentrations in the liquid after plasma treatment. The acidic conditions in the water caused by plasma using air as the working gas is likely to be due to nitric acid production, where nitrogen and oxygen react with water to form the following acids:

1 $${{\mathrm{NO}}_{2}}^{-}+{\mathrm{H}}^{+}\to {\mathrm{HNO}}_{2}$$

2 $${\mathrm{NO}}_{2}+\mathrm{OH }\to {\mathrm{HNO}}_{3}$$

3 $${{\mathrm{NO}}_{2}}^{-}+{\mathrm{H}}_{2}{\mathrm{O}}_{2}+ {\mathrm{H}}^{+}\to \mathrm{N}{{\mathrm{O}}_{3}}^{-}+{\mathrm{H}}_{2}\mathrm{O }{+\mathrm{ H}}^{+}$$

**Figure 1:**
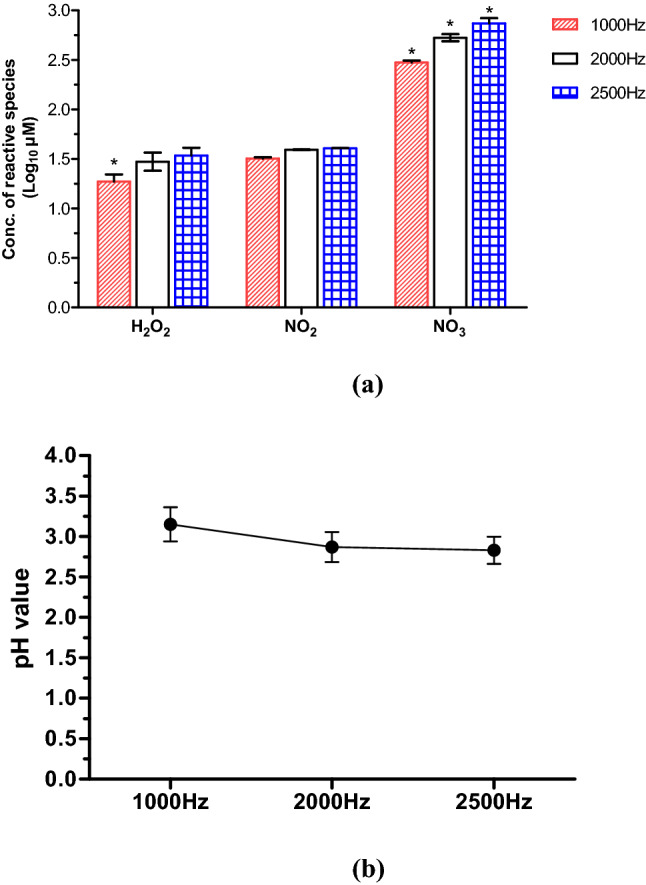
(**a**) Concentration of reactive oxygen/nitrogen species (RONS) and (**b**) pH values measured in water treated with a plasma pin-reactor at different system frequencies for a treatment time of 5 min. Error bars indicate standard deviation. Data were collected from two independent experiments, each one performed in duplicate (n = 4). Asterisk (*) represents significant difference (p ≤ 0.05) between different plasma system frequencies for each reactive species (H_2_O_2_ or NO_2_ or NO_3_). Graphs were generated using GraphPad Prism version 5.0 (GraphPad Software, San Diego, California USA, http://www.graphpad.com).

As shown in Fig. 1, high concentrations of nitrate were detected after 5 min of plasma treatment; moreover, the concentrations were further increased at the higher frequency of 2500 Hz. Additionally, small concentrations of hydrogen peroxide and nitrite were also detected. Owing to the longer treatment and acidic conditions, nitrite can degrade and transform into nitrates^17^. Moreover, hydrogen peroxide reacts with nitrites to form nitrates or peroxynitrites^17^. No significant difference was observed in hydrogen peroxide and nitrites detected at different plasma treatment frequencies, except in the case of nitrates.

Depending on the plasma characteristics, higher frequency can significantly increase discharge current, power, gas conductivity which in turn could increase reactive species in a sample^18,19^. In this study, higher frequency resulted in a higher concentration of nitrates. Plasma generated reactive species are important factors in cold plasma treatment; they act synergistically with acidic pH, create favourable conditions for the penetration of RONS into the cell membrane^20^. It would therefore be expected that increasing the frequency (in this case) would influence bacterial inactivation efficacy, yielding improved plasma-mediated biofilm eradication. Therefore, a constant operating frequency of 2500 Hz was chosen for all further experiments.

### Analysis of intracellular reactive species generated by atmospheric air plasma treatment

**Figure 2:**
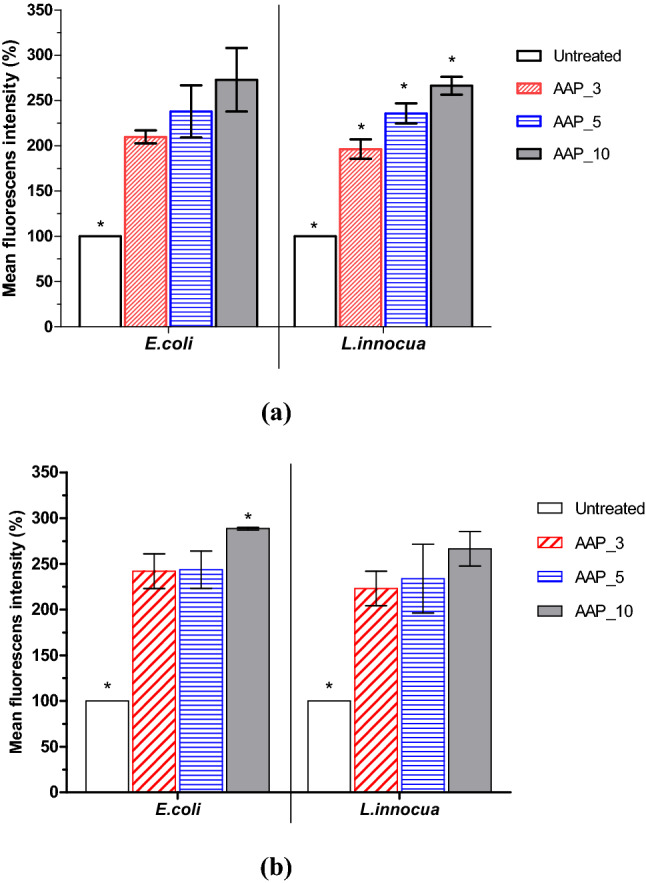
Intracellular levels of RONS in *E. coli* and *L. innocua* 96 h biofilm cells after atmospheric air plasma treatment (**a**) ROS (**b**) RNS levels. Data are the mean percentage values of fluorescence intensity. Results represent the average of two independent experiments, each one performed in triplicate (n = 6). Statistical analysis and graphs were generated using GraphPad Prism version 5.0 (GraphPad Software, San Diego, California USA, http://www.graphpad.com). Separate one-way ANOVA tests were performed for each biofilm forming species to make a comparison between the untreated control and the different atmospheric air plasma treatments. Asterisk indicates significance difference (p ≤ 0.05). AAU_15: airborne acoustic ultrasound treatment for 15 min; AAU_30: airborne acoustic ultrasound treatment for 30 min; AAP_3: atmospheric air plasma treatment for 3 min; AAP_5: atmospheric air plasma treatment for 5 min; AAP_10: atmospheric air plasma treatment for 10 min.

Biofilm has complex defence systems that help protect embedded cells in the EPS to maintain homeostasis and help protect cells located deeper within the matrix from disinfectants by permitting only limited diffusion of chemicals. Therefore, this study also investigated reactive oxygen and nitrogen species (RONS) within biofilm cells post AAP treatment. Data shown in Fig. 2 represents the intracellular RONS concentrations generated after AAP treatment expressed as mean fluorescence intensity (%). The results show that AAP-exposed cells had significantly augmented DCFH-DA and DAF-FM DA intensity, indicating significant increases in intracellular RONS levels. In particular, after 5 min of plasma exposure, ROS levels and RNS levels were more than twice that of the untreated controls. The gradual rise in the DCF and DAF intensity suggests the accumulation of intracellular ROS and RNS in biofilm cells with increasing AAP treatment. Previous studies have also found significant (P < 0.05) increases in intracellular ROS in *Listeria monocytogenes* (EGD-e) cells after dielectric barrier discharge cold plasma treatments^21^. Long plasma treatment produced higher reactive species which caused oxidative stress in *L. monocytogenes* cells, which was indicated by higher intracellular ROS levels. The study also demonstrated significant up-regulated oxidative stress response genes with increasing plasma treatments. The combined interaction of plasma constituents, electric fields, ions and electrons, along with biological cellular components could invoke oxidative responses in bacteria, inducing many detrimental effects on biofilms.

### Effect of atmospheric air plasma treatment and airborne acoustic ultrasound treatment on cultivability and metabolic activity of bacterial biofilms

**Figure 3:**
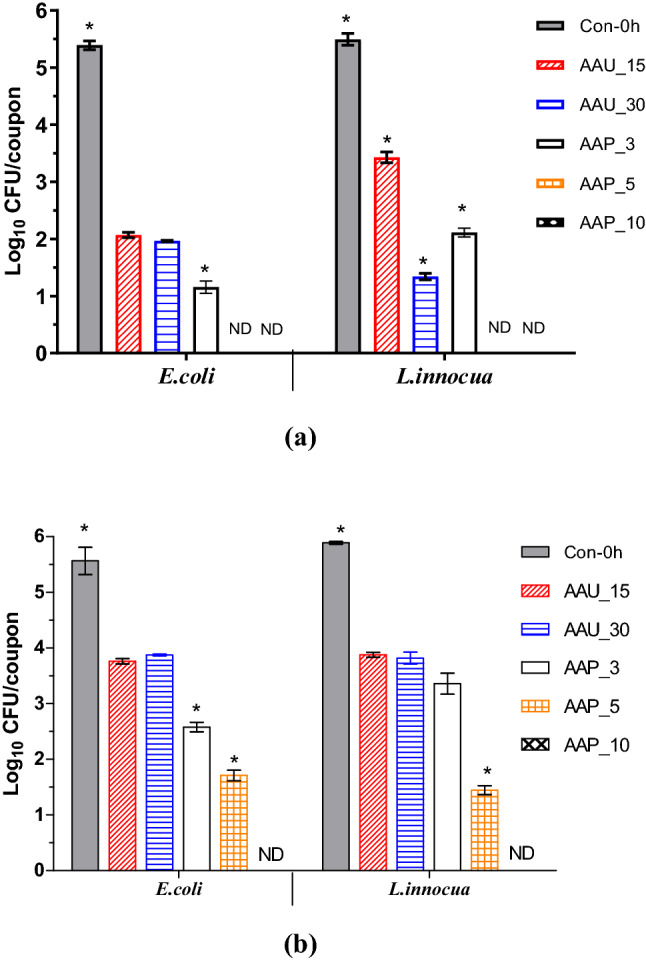
Inactivation efficacy of airborne acoustic ultrasound and atmospheric air plasma technology against (**a**) 48 h and (**b**) 96 h *E. coli* and *L. innocua* biofilms. ND: not detected, below detection limit. Vertical error bars indicate standard deviation (n = 4). Asterisk indicates significant differences (p ≤ 0.05) between different treatments for each bacterial strain, analysed by one-way ANOVA and Tukey multiple comparison post hoc test. Graphs were generated using GraphPad Prism version 5.0 (GraphPad Software, San Diego, California USA, http://www.graphpad.com). AAU_15: airborne acoustic ultrasound treatment for 15 min; AAU_30: airborne acoustic ultrasound treatment for 30 min; AAP_3: atmospheric air plasma treatment for 3 min; AAP_5: atmospheric air plasma treatment for 5 min; AAP_10: atmospheric air plasma treatment for 10 min.

To evaluate the abilities of atmospheric air plasma and airborne acoustic ultrasound technology to inactivate bacterial biofilms, *E. coli* and *L. innocua* biofilms were exposed to AAP or AAU for different treatment times. The plate count assay indicated the bactericidal effect of both AAP and AAU treatments (Fig. 3). Both treatments significantly reduced *E. coli* and *L. innocua* biofilm populations. An AAP treatment of 5 min was able to inactivate 48 h biofilm cells below the detection limit (l log CFUcm^2^), while airborne acoustic treatment of 30 min reduced the *E. coli* and *L. innocua* counts by 3.42 and 4.05 log_10_ CFU/coupon, respectively. The maturity of the biofilm significantly influenced the antimicrobial efficacy of the atmospheric air plasma and airborne acoustic ultrasound process. For 96 h biofilms, it was observed that both the non-thermal treatments were less effective than for the 48 h biofilms. This could be explained by the fact that mature biofilms are denser in biofilm mass than younger ones, limiting diffusion of antimicrobial agents into the biofilm matrix^22^. Longer AAP treatment periods were required for the 96 h biofilm cells to reduce the bacterial population to undetectable levels. As observed with intracellular RONS results, increasing plasma treatment time, there were more reactive species produced inside the cell. The longer treatment time allowed more reactive plasma species to penetrate the biofilm matrix, interact with the cells and oxidize them, resulting in a stronger inactivation effect, even with increased biofilm age. Similarly, Soler-Arango et al.^23^ found a strong bactericidal effect of gas discharge plasma treatment that reduced the concentration of the *Pseudomonas aeruginosa *biofilms by 3.4 log CFU/mL after 3 min of plasma exposure and complete deactivation (˃ 99.999% killing efficacy) after 15-min plasma treatment.

In the case of ultrasound treatment, no significant changes were evident between 15- and 30-min treatments. Previous research by Bi et al.^24^ found that inactivation of *Salmonella typhimurium* cells was relatively rapid for an initial treatment period of 20 min with a contact-type ultrasound treatment, but was followed by a tailing effect for longer treatments. The resistance of *E. coli* and *L. innocua* biofilms towards AAU treatment could be due to the protective nature of the biofilm’s structure. Overall, the bactericidal effects of atmospheric air plasma on *E. coli* and *L. innocua* were greater than that of airborne acoustic ultrasound in both early and mature biofilm cultures.

**Figure 4:**
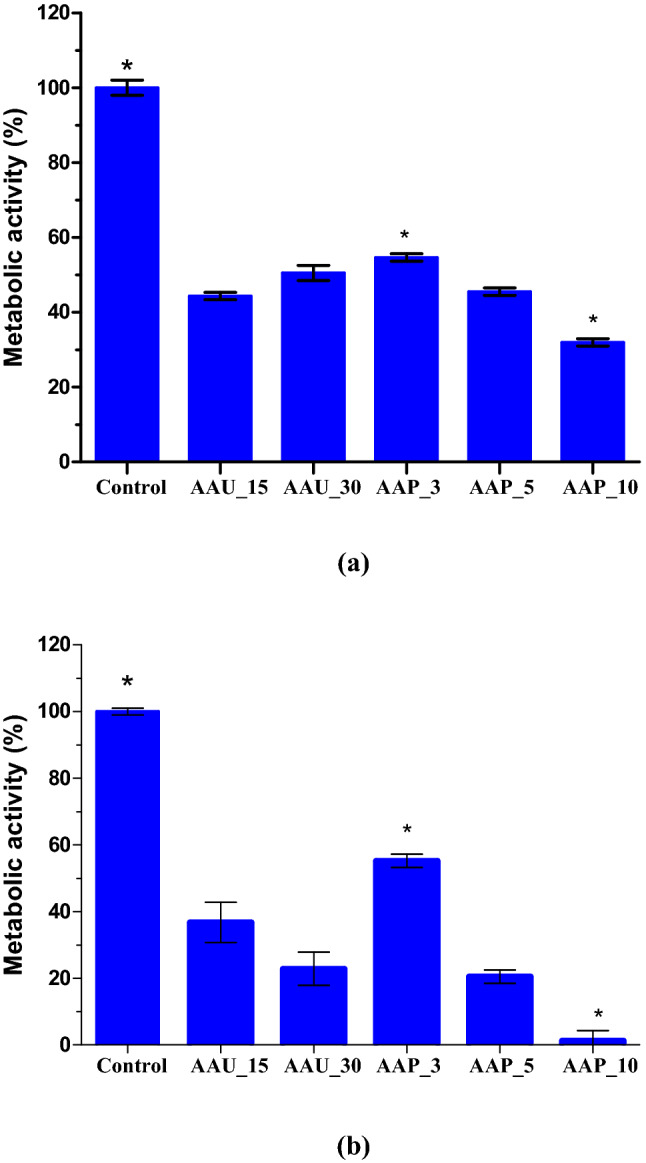
Effect of airborne acoustic ultrasound and atmospheric air plasma treatment on metabolic activity of 96 h (**a**) *E. coli* and (**b**) *L. innocua* biofilms. Error bars indicate standard deviation (n = 4). Significant differences between different treatments are marked by asterisks (P < 0.05; One-way ANOVA test and Tukey multiple comparison post hoc test). Graphs were generated using GraphPad Prism version 5.0 (GraphPad Software, San Diego, California USA, http://www.graphpad.com). AAU_15: airborne acoustic ultrasound treatment for 15 min; AAU_30: airborne acoustic ultrasound treatment for 30 min; AAP_3: atmospheric air plasma treatment for 3 min; AAP_5: atmospheric air plasma treatment for 5 min; AAP_10: atmospheric air plasma treatment for 10 min.

As air plasma and airborne acoustic ultrasound treatment affect biofilm, they may impact enzymatic activity associated with the metabolic activity of the cell. The effects of both AAP and AAU treatments on the metabolic activity of 96 h *E. coli* and *L. innocua* biofilm cells were evaluated by a calorimetric method using the XTT assay. As shown in Fig. 4, 3 min AAP exposure resulted in a 45% reduction in metabolic activities of both *E. coli* and *L. innocua* cells. In the case of bacterial cells exposed to 15 min AAU, the metabolic activity of *E. coli* and *L. innocua* cells were reduced by 55% and 63%, respectively. The metabolic activity of *E. coli* and *L. innocua* cells demonstrated a gradual reduction with increasing AAP treatment, while there was no significant difference observed between 15 and 30 min for AAU treated cells. This is in agreement with the culturable plate count data. Biofilm cells treated with 10 min AAP, which were below the detection limit as measured by the plate count method, indicated metabolic activity. These results suggest the presence of viable-but-non-culturable (VBNC) or persister cells; where cells are dormant, slow-growing (that were below detection limit by plate count assay), or in a non-growing state, which was also referred to by Gilmore et al.^25^ and Ziuzina et al.^26^. The VBNC state generally occurs when bacteria are under stress conditions such as oxidative stress, starvation, acidic, osmotic and other adverse conditions.

### Effect of atmospheric air plasma and airborne acoustic ultrasound on viability, membrane integrity and bacterial morphology of biofilm cells.

**Figure 5:**
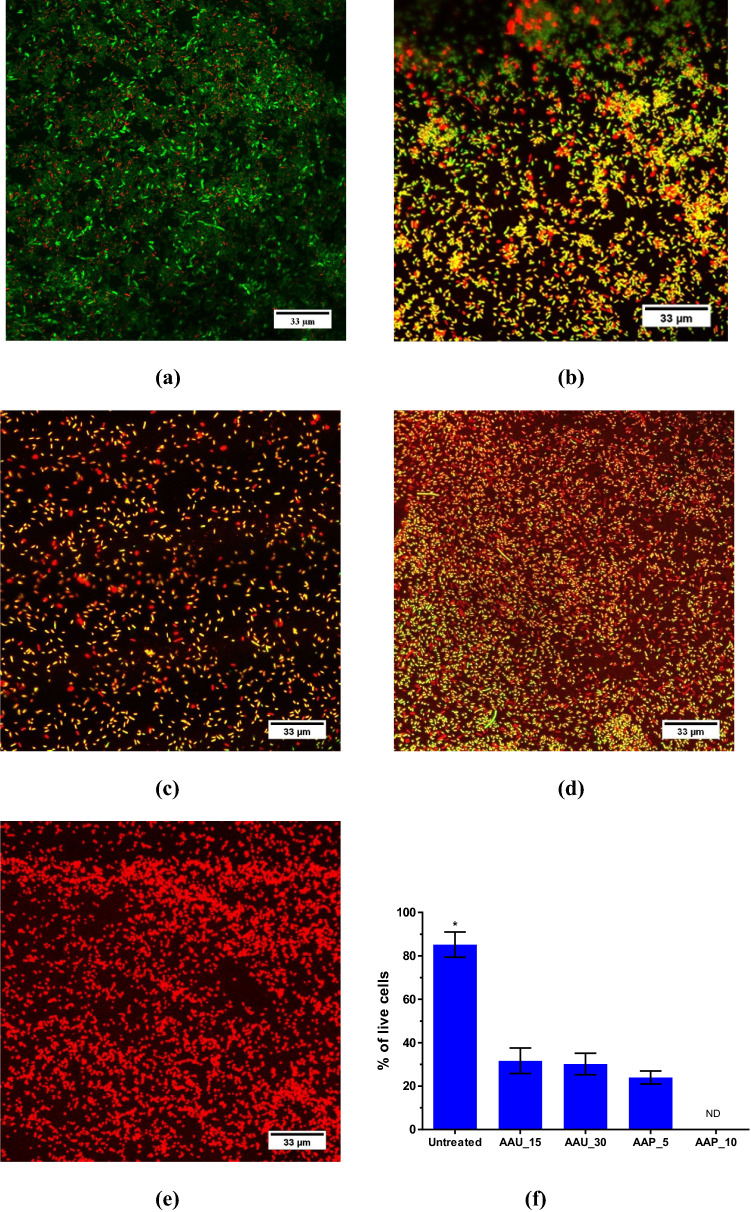
(**a**–**e**) Confocal laser scanning microscopy images of *E. coli* 96 h biofilm stained with Live/Dead stain (**a**) untreated control; (**b**) 15 min AAU treatment; (**c**) 30 min AAU treatment; (**d**) 5 min AAP treatment; (**e**) 10 min AAP treatment. Scale bars = 33 μm. SYTO 9 (green cells) denotes live viable cells, whereas the propidium iodide (red/yellow cells) indicates dead cells that have been deactivated because of ruptured or damaged membranes; (**f**) percentage of live viable cells after AAU and AAP treatments (n = 6). The asterisk indicates significance difference between different treatments (p ≤ 0.05) analysed by one-way ANOVA and Tukey multiple comparison post hoc test. Graph was generated using GraphPad Prism version 5.0 (GraphPad Software, San Diego, California USA, http://www.graphpad.com).

To evaluate the effect of AAP and AAU on the viability of cells embedded in the biofilm, *E. coli* biofilm cells were stained with SYTO 9 and propidium iodide (PI) for examination by fluorescence Live/Dead assay and confocal laser scanning microscopy (CLSM). The confocal images acquired from SYTO/PI stained *E. coli* biofilms before and after treatment are displayed in Fig. 5a–e. The untreated biofilm cells were predominantly green, indicating that a large number of cells present were live cells. With AAU and AAP treatment, green live cells diminished gradually, and the fraction of red dead cells increased, indicating that the vast proportion of the biofilm cells were dead which further corroborated the findings obtained from the plate count experiments. The fluorescence Live/Dead assay, which was used to quantify the viability of the cells after AAP and AAU treatment, also showed a decline in live cells (Fig. 5f). After AAU treatment (15- and 30- min), a large number of cells were dead; the percentage of live cells were reduced to 31 and 33%. As the plasma processing time increased, the percentage of live cells with intact membranes decreased in a plasma dose-dependent manner.

**Figure 6:**
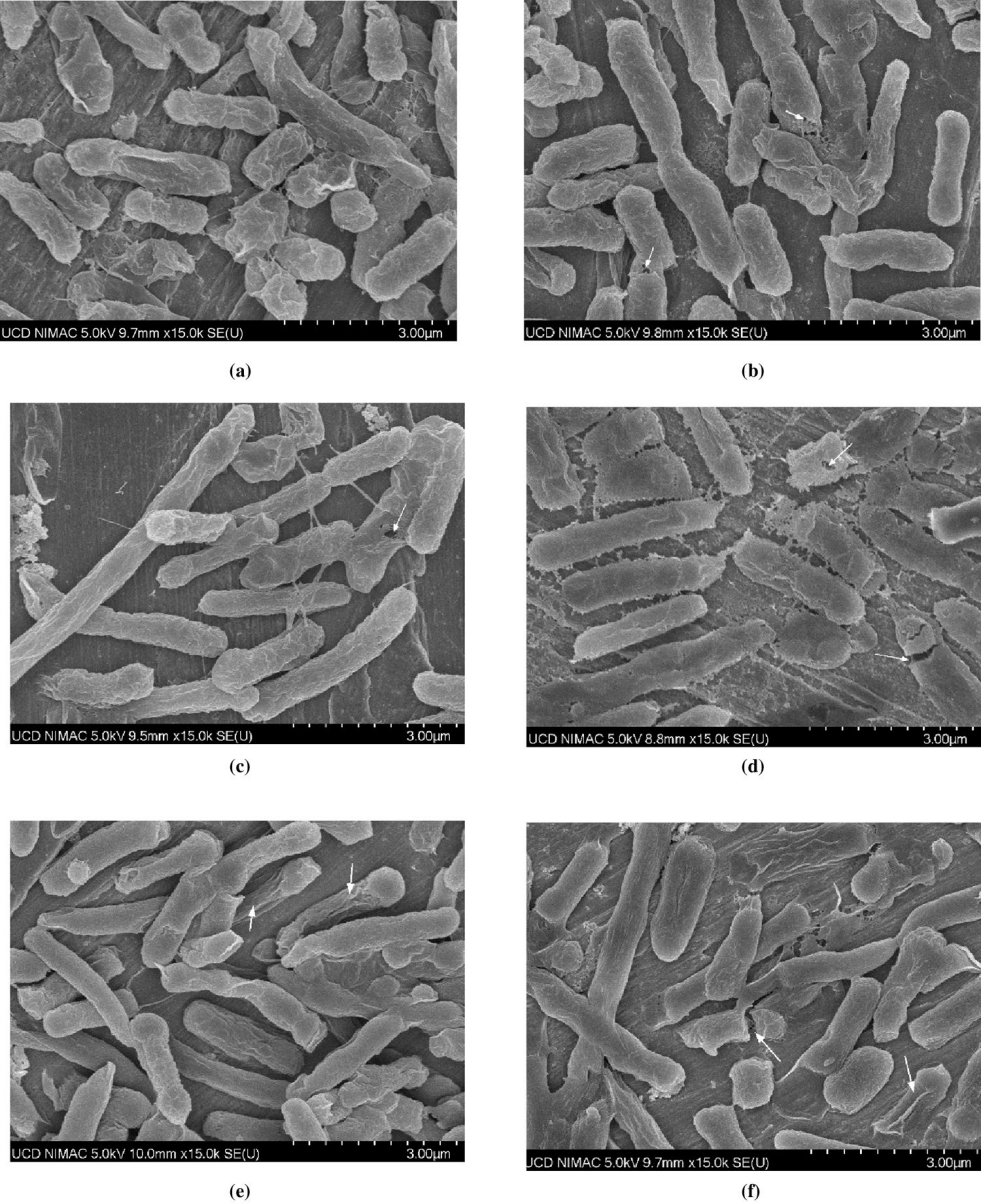
Scanning electron micrographs of 96 h *E. coli* biofilm—(**a**) Untreated control; (**b**) 15 min AU treatment; (**c**) 30 min AAU treatment; (**d**) 3 min AAP treatment; (**e**) 5 min AAP treatment; (**f**) 10 min AAP treatment. White arrows indicate visibly deformed structures and pores.

In order to gain a deeper understanding of the plasma/ultrasound-induced changes in bacterial morphology and cell injury, SEM analysis was performed on *E. coli* biofilms (Fig. 6). The untreated samples revealed a large amount of rod-shaped *E. coli* cells with interspersed extracellular polymeric substance (EPS) observed on cells and between cells and the coupon surfaces. Compared to the untreated controls, there were considerable morphological alterations and structural damages observed post-treatment. After AAU and AAP treatment a large proportion of *E. coli* cells were disintegrated, and cell debris was found on the surface. Furthermore, some *E. coli* cells showed pore formation and localised ruptures after treatment. This indicates both treatments physically affected the biofilm and cell structure of the bacteria. From the morphology study, the maximum physical effects were observed after 30 min of AAU and 5–10 min of AAP treatment. In the SEM images (Fig. 6e), the majority of the EPS layer was removed after 5 min of plasma treatment. Notably, in some areas, the bacterial cells appeared rough but still intact. It should be noted that an undamaged cell morphology after treatment does not signify a viable cell. In addition to morphological damage, cold plasma or ultrasound treatment also affect intracellular components, including DNA, protein, as well as enzymes.

The physical effects of AAP have been well recognised as one of the major modes of bacterial inactivation^20,27^. Although cold plasma treatment can kill bacteria, damage biofilm matrix and affect EPS, the mechanisms underlying its effects are unclear. The ROS/RNS can penetrate cells and are known to cause oxidative and nitrosative damage to lipids and protein in bacteria. This damage induces lipid peroxidation, inhibition of enzyme functions and alteration of DNA, which ultimately accounts for bacterial cell death^27^. The chemical reaction between reactive species and acidification of the milieu, combined are responsible for the lethality to the microorganism^20^. Previous studies by Los et al.^28^ and Patange et al.^9^ evaluated the disinfection potential of acidified (by HCl) solutions compared to plasma treatment on bacterial biofilm and spores. The study reported a minimum or reduced antimicrobial effect of acidified water compared to plasma treatment. Kondeti et al.^29^ reported acidified hydrogen peroxide solution at the same pH as cold plasma solution had no inactivation effect on *Pseudomonas aeruginosa* and *Staphylococcus aureus*. Similarly, Schnabel et al.^30^ compared inactivation efficacy between plasma treatment and a nitric acid solution; the results demonstrated comparable inactivation rates only with a very strongly acidified HNO_3_ solution. While pH supports inactivation, the combined interaction of plasma constituents and acidification causes oxidative stress in bacteria and its intracellular components, ultimately inducing adverse effects on biofilms.

In the case of AAU, the rapid series of contraction and expansion of acoustic pressure are speculated to deliver the observed mechanical effects on bacteria^11^. Absorption of acoustic energy by the bacteria, may alter the cell morphology and disrupt cell membrane integrity. The high acoustic pressure could also enhance ultrasonic wave stress-causing pores in cell membranes, eventually causing cell death. There have been numerous studies examining the antimicrobial efficacy of ultrasound in food^12^; however, most of these studies employed contact-based ultrasound treatment. The acoustic intensity used in this study was significantly low. The exact mechanism of microbial biofilm inactivation by non-contact type ultrasound is not yet fully understood and needs to be further explored.

### Comparison of the inactivation efficacy of sodium hypochlorite, atmospheric air plasma and airborne acoustic ultrasound system against *E. coli* biofilm

**Figure 7:**
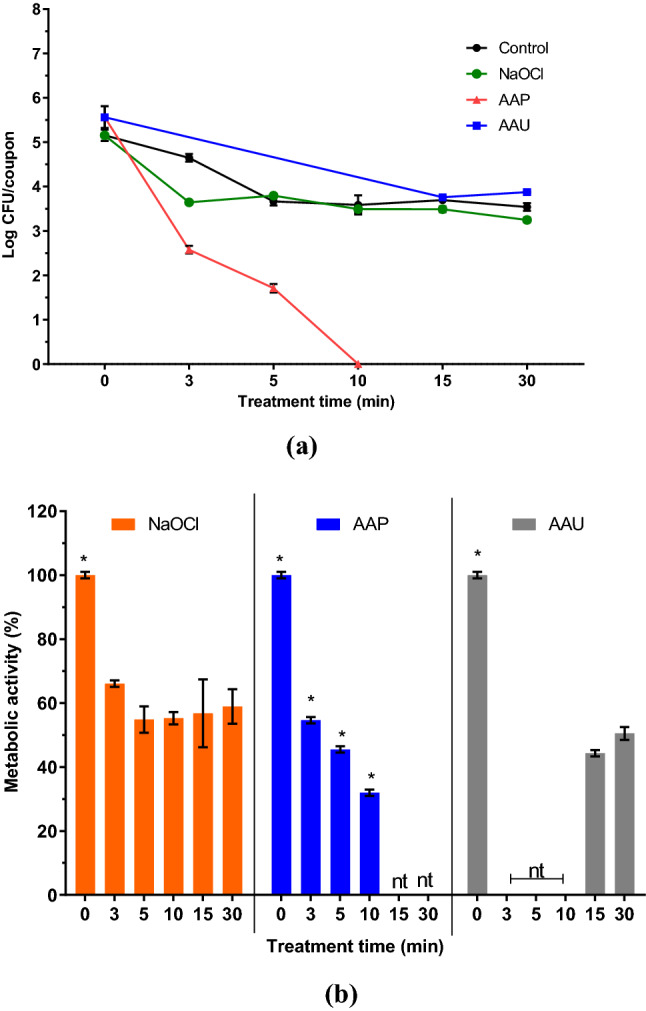
Effects of sodium hypochlorite (NaOCl; 200 ppm), atmospheric air plasma and airborne acoustic ultrasound treatments on 96 h *E. coli* biofilms—effect on (**a**) culturability (Log CFU/coupon) and (**b**) metabolic activity (%). Error bars show the standard deviation (n = 4). nt = not treated. The asterisk mark indicates significance difference between different treatments (p ≤ 0.05) analysed by one-way ANOVA and Tukey multiple comparison post hoc test. Graph was generated using GraphPad Prism version 5.0 (GraphPad Software, San Diego, California USA, http://www.graphpad.com).

An additional experiment was performed to compare the antimicrobial effect of atmospheric air plasma, airborne acoustic ultrasound treatment with a commonly used disinfectant such as sodium hypochlorite. Chlorine and chlorine-based products are routinely used as disinfection agents in the food industry and health care sectors for cleaning surfaces, equipment, fresh produce and food contact surfaces^31–33^. The effects of the chlorine solutions on 96 h *E. coli* biofilms from SS coupons are shown in Fig. 7. The biofilm population showed a significant reduction of 1.5 Log CFU/coupon and a 33% reduction in metabolic activity with an initial 3 min NaOCl treatment (200 ppm), compared to untreated controls. However, no further significant inhibition was observed in biofilm cells exposed to longer NaOCl treatments (5–30 min); the bacterial population remained between 3.6 and 3.2 Log CFU/coupon, suggesting no further killing of cells. A similar phenomenon was observed with the metabolic activity of the biofilm cells exposed to NaOCl for a longer treatment time. With the technology-based treatments, a greater bactericidal effect was observed with AAP when compared to NaOCl, whereas AAU showed similar effects to NaOCl.

In order to maintain chlorine disinfection efficiency, the food industry has to constantly maintain the active chlorine concentration. The continuous addition of chlorine and its reaction with organic components found in the food industry can produce chlorine by-products, which have potential carcinogenic and mutagenic compounds^34,35^. Furthermore, studies have reported the capability of microorganisms to develop resistance to chlorine-based treatment, where microbes can survive and grow despite biocidal treatments^36,37^. These findings demonstrate the great potential of AAP and AAU for the elimination of bacterial biofilms and their suitability for substituting for the more traditional chlorine-based treatments used in the food industry and healthcare sectors.

## Conclusion

Based on findings from this study, it can be concluded that both atmospheric air technologies assessed demonstrated biofilm cell inactivation effects for *E. coli* and *L. innocua* biofilms. Atmospheric air plasma treatment was more effective for both early and mature stage biofilms than the airborne acoustic ultrasound treatment. Treatment for 5 min by AAP significantly reduced *E. coli* and *L. innocua* biofilm cell counts by 3.5 to 4.5 Log CFU/coupon, as well as reducing the metabolic activity by 54%. This could be mainly attributed to increased intracellular RONS observed in the treated cells, causing oxidative stress within the cells and consequently accelerating death of the bacterial cells. The results obtained from SEM and CLSM indicated that both AAU and AAP affected the biofilm cell morphology and cell viability. The degree of damage and inactivation obtained with this study suggest the potential to improve or optimise disinfection strategies using a combination of airborne acoustic ultrasound and atmospheric air plasma treatments against complex bacterial biofilms. However, more investigation is necessary to have a better understanding of the reactive species’ distribution, penetration and the inactivation mechanisms of atmospheric air plasma and airborne acoustic ultrasound technologies for future real-world applications.

## Materials and methods

### Bacterial strains and culture conditions

*Escherichia coli* K-12 ER2925 and *Listeria innocua* NCTC 12,210 strains were used in our experiments, which were provided by Dr. Des Walsh from the microbiology culture collection at the Teagasc Food Research Centre. Stock cultures were maintained at − 80 °C, streaked onto tryptone soya agar CM0131 (TSA; Oxoid Limited, Ireland) and incubated at 37 °C for 24 h. Individual cultures were transferred to 10 ml of tryptone soya broth CM0129 (TSB; Oxoid Limited, Ireland) and incubated at 37 °C for 18 h. After incubation, each culture was washed three times with 10 mL of phosphate buffered saline BR0014 (PBS; Oxoid Limited, Ireland) at 7000×*g* for 6 min, re-suspended in 10 ml of TSB and the concentration standardised to approximately 1 × 10^7^ CFU/coupon.

### Preparation of coupons and biofilm formation

The biofilms were cultured on stainless steel (SS) coupons grade 304 (Watermark Engineering, Dublin, Ireland) of 15 mm diameter and 1.2 mm thickness. The coupons were sterilised by autoclaving at 121 °C for 15 min. For confocal laser scanning microscopy (CLSM) analysis, *E. coli* biofilms were produced on a glass coupon (1 × 1 cm). To eliminate the presence of any residues on the glass coupons which could affect the microscopy data, the glass coupons were acid washed with 1 M HCl followed by 70% ethanol, as described by Fischer and co-workers^38^. The glass coupons were air dried in a fume hood and autoclaved at 121 °C for 15 min.

For biofilm formation on both SS and glass, 3 ml of a standardised cell suspension was dispensed in each well of 12 well plates containing the sterilised coupons. The inoculated coupons were incubated for 48 h and 96 h at 37 °C in an orbital shaker. After every 24 h incubation the TSB medium was replaced with equal volumes of fresh medium and incubation continued under the same conditions. To remove non-adherent cells, coupons were gently washed with 3 ml of PBS. The coupons were then placed in a bio-safety cabinet to dry for 20–30 min before plasma or acoustic treatment.

### Treatment of biofilms with atmospheric air plasma or acoustic airborne ultrasound technology

#### Atmospheric air plasma treatment (AAP)

**Figure 8:**
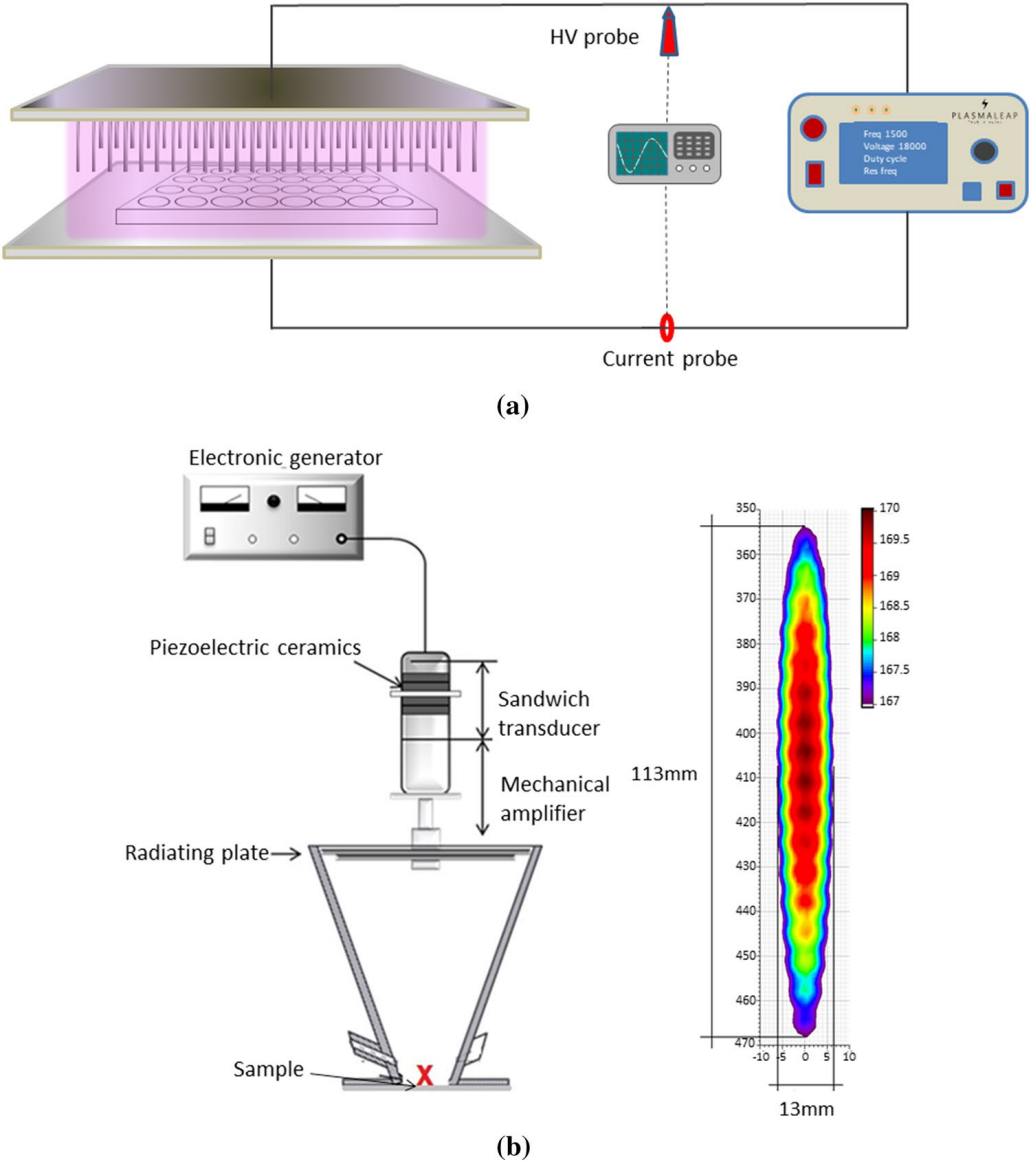
Schematic diagram of bacterial coupons treated with (**a**) atmospheric air plasma or (**b**) acoustic airborne ultrasound system and its intensity distribution.

The atmospheric air plasma used in this study was a multi-pin (11 × 8) plasma system (Leap100, PlasmaLeap Technologies, Dublin, Ireland). The discharge is generated between the ends of the high voltage pins and the flat ground base plate. The discharge gap was set to 55 mm. Plasma was generated in the discharge gap using atmospheric air at varying discharge frequencies of 1000 Hz, 2000 Hz and 2500 Hz, at a fixed discharge voltage of 40 kV. The samples were treated with AAP for different treatment times ranging between 0 and 10 min. Either 96-well micro-titre plates containing 200 µl of deionised water treated for reactive oxygen and nitrogen species (RONS) analysis, or petri dishes containing SS and glass coupons were placed directly between the electrodes for treatment. The lid of the container was removed during the plasma treatment process. Figure 8a shows a schematic diagram of the bacterial coupon treatments using the multi-pin plasma system.

RONS analysis was carried out to investigate the optimal treatment parameters with the plasma system. The chemical composition and the pH were examined following 5 min of AAP treatment. Reactive species including hydrogen peroxide, nitrite and nitrate species generated by the plasma system in plasma treated water were analysed using a titanium oxysulfate calorimetric method, Griess reagent (N-(1-naphthyl) ethylenediamine dihydrochloride) and 2,6-dimethyl phenol (DMP) Spectroquant nitrate assay kit, as previously described by Lu et al.^39^. The pH of the treated water was measured using a SevenCompact pH meter (S210; Mettler-Toledo GmbH, Switzerland).

#### Airborne acoustic ultrasound treatment (AAU)

Airborne acoustic ultrasound treatments were carried out using an airborne ultrasonic system (Pusonics S.L., Madrid, Spain). The system was operated at a frequency of 26 kHz, with a maximum power output of 170 W. A detailed description of the system has been previously described by Charoux et al.^40^. The biofilm coupons were placed on a stainless-steel plate at 42.5 cm from the focus centre of the transducer, where the maximum acoustic energy density of 10 W/cm^2^ was located (Fig. 8b). The biofilm coupons were treated with airborne acoustics for 15 and 30 min.

### Quantification of intracellular reactive species generated by atmospheric air plasma treatment

The production of intracellular RONS in the biofilms was measured using 2′,7′-dichlorofluorescin diacetate (DCFH-DA; Sigma-Aldrich, Arklow, Ireland) assay and 4-amino-5-methylamino- 2′,7′-difluorofluorescein diacetate (DAF-FM DA; Sigma-Aldrich, Arklow, Ireland) assay, as previously described by Han et al.^41^, with minor modifications. Each treated or non-treated *E. coli* biofilm coupon was vortexed for 2 min with 10 ml of 0.85% NaCl solution and 10 glass beads (18406, Sigma-Aldrich, Arklow, Ireland). This bacterial suspension was used for the detection of intracellular RONS.

DCFH-DA was used to detect intracellular ROS, which could passively diffuse through the cell membrane and form non-fluorescent DCFH. The DCFH reacts with ROS to form fluorescent 2′,7′-dichlorofluorescein (DCF), which is trapped within the cell and measured using a fluorescence spectrophotometer. Treated and non-treated bacterial suspensions were incubated with DCFH-DA at a final concentration of 5 μM in PBS for 15 min at 37 °C in the dark. Incubated aliquots of 200 μl were transferred into 96-well fluorescence micro-plates (Costar Multiple Well Cell Culture Plates, Fisher Scientific Ireland Ltd) and measured at excitation and emission wavelengths of 485 nm and 530 nm using a Spark multimode microplate reader (Tecan UK Ltd. UK).

DAF-FM DA was used to quantify intracellular RNS levels. DAF-FM DA is a cell permeable reagent, which is hydrolysed to 4-amino-5-methylamino-2′,7′-difluororescein (DAF-FM) by intracellular esterase and reacts with NO inside the cell to yield benzotriazole which is highly fluorescent. AAP treated and non-treated *E. coli* cells were incubated with DAF-FM DA at a final concentration of 1 μM in PBS for 15 min at 37 °C in the dark. Samples were then transferred into new 96-well fluorescence microplates and measured at excitation and emission wavelengths of 490 nm and 515 nm. Each sample was read in three wells and replicated twice. The intracellular RONS were measured immediately after AAP treatment. The percentage of mean fluorescence intensity was calculated by comparing the absorbance of the treated samples with the absorbance of the untreated control samples.

### Determination of cell viability and metabolic activity

The viable counts in the biofilms on coupons before and after treatment were determined via plate counts. Untreated controls or treated biofilm SS coupons were placed in a tube containing 10 ml of maximum recovery diluent (MRD; Oxoid Ireland c/o Fannin Healthcare, Ireland) and 10 glass beads and vortexed for 2 min to disrupt any biofilm present on the coupons’ surface. The resulting bacterial suspensions were serially diluted and plated on TSA plates and incubated for 24 h at 37 °C. Quantitative analysis was performed in duplicate for each experiment. The total viable count was determined by standard colony counts and expressed as logarithmic colony forming units (Log_10_ CFU/coupon).

The metabolic activity of the bacterial cells before and after plasma and airborne acoustic treatments were determined by the XTT assay (2,3-bis(2-methoxy-4-nitro-5-sulfophenyl) [phenyl-amino) car-bonyl]-2H-tetrazolium hydroxide; XTT) as previously described by Peeters et al.^42^, with a few modifications. A XTT stock solution was prepared by adding 4 mg XTT (Sigma Aldrich, Arklow, Ireland) to 1 ml of pre-warmed 1× PBS. This solution was supplemented with 100 µl of menadione solution (containing 5.5 mg/L menadione (Sigma Aldrich, Arklow, Ireland) in acetone). The untreated/treated biofilm coupons were transferred to new 12-well tissue culture plates, and 300 µl of freshly prepared XTT–menadione solution and 300 µl of 1× PBS were placed on each biofilm coupon. After 5 h incubation at 37 °C in the dark, the medium was removed and dispensed in sterile Eppendorf tubes. The medium was centrifuged for 5 min at 6000×*g*. For spectrophotometric readings, 100 µl of supernatant was transferred to new 96-well microtiter plates and absorbance was read at 490 nm. Untreated biofilm coupons were used as the control. The percentage of metabolic active cells was calculated by comparing the absorbance of the treated samples with the absorbance of the untreated control samples.

### Bacterial staining for confocal laser scanning microscopy (CLSM) and fluorescence Live/Dead assay

Biofilms before and after treatment by AAP and AAU were stained using the Live/Dead BacLight Bacterial Viability kit L7007 (Thermofisher Scientific, Dublin, Ireland) according to the manufacturer’s protocols.

For confocal analysis, a fresh working solution was prepared by adding 1.5 μl of each SYTO 9 and PI stain to 1 ml of 0.85% sterile sodium chloride (NaCl) solution. One hundred microliters of staining solution was added onto each biofilm coupon and incubated at room temperature in the dark for 15 min. The coupons were washed gently with NaCl twice to remove excess stain and its residues. The stained samples were placed in 35 mm diameter glass-bottomed µ-dish (ibidi GmbH, Martinsried, Germany) with a drop of mounting solution from the staining kit. Biofilms were visualised and imaged using a confocal laser scanning microscope (Olympus Fluoview FV1000) equipped with a 60×/1.35 NA oil immersion objective.

For microplate reader analysis, bacteria were re-suspended from the coupons’ surface in 10 mL of sterile water using glass beads as described previously. Samples were stained by adding 100 μl freshly prepared stain solution (3 μl SYTO 9 + 3 μl PI working + 1 ml 0.85% NaCl solution) to 100 μl bacterial suspension per well in a 96-well fluorescence micro-plate (Costar Multiple Well Cell Culture Plates, Fisher Scientific Ireland Ltd). The stained samples were incubated at room temperature in the dark for 15 min. Fluorescence intensity was measured with the Spark multimode microplate reader (Tecan UK Ltd. UK) using a 488 nm excitation filter (for both SYTO9 and PI) and a 530 nm (for SYTO 9) and 630 nm (for PI) emission filter.

### Scanning electron microscope (SEM)

SEM samples were prepared as previously described by Patange et al.^9^, with minor modifications. Following exposure to plasma or airborne acoustic treatment, SS coupons were fixed with 2.5% glutaraldehyde (Sigma-Aldrich, Arklow, Ireland) in 0.05 M sodium cacodylate buffer (SCB; Fisher Scientific Ireland Ltd, Dublin, Ireland) (pH 7.4) for 2 h at 4 °C. After fixing, coupons were washed with SCB three times and fixed in 1% osmium tetroxide (Sigma-Aldrich, Arklow, Ireland) for 2 h at 4 °C. The coupons were then rinsed once with SCB and twice with sterile distilled water. The fixed coupons were dehydrated using an increasing series of ethanol (30, 50, 70, 80, 95 and 99.5%) for 5 min each and 15 min in 99.5%. The coupons were then further dehydrated through a series of 33%, 50%, 66%, and 100% of hexamethyldisilazane (HMDS; Sigma-Aldrich, Arklow, Ireland) prepared in 99.5% ethanol. The coupons were left to dry overnight in a fume hood. Samples were coated with gold using a Q150T ES sputtering device (Quorum Technologies Ltd, United Kingdom) and examined using a field emission scanning electron microscope (FE-SEM SU8240, Hitachi High Technologies, USA).

### Treatment of biofilms with sodium hypochlorite

The working solution was prepared by diluting 12.5% sodium hypochlorite (VWR International Ltd., United Kingdom) in distilled water to obtain a final concentration of 200 ppm NaOCl. The prepared 96 h *E. coli* biofilm coupon was immersed in 3 ml NaOCl solution and sterile distilled water (control) for 3, 5, 10, 15 and 30 min. After treatment, samples were immediately neutralized by using 3 ml sodium thiosulfate (0.1 M) solution. The sample was then analysed by plate count and XTT assay as described before.

### Statistical analysis

All experiments were carried out in duplicate and the data presented as means ± standard deviations. Graphpad Prism version 5.0 (GraphPad software, San Diego, California, USA) was used for graphic representation and statistics calculations. One-way (ANOVA) followed by Tukey multiple comparison post hoc test was applied to evaluate the antimicrobial effect of the treatments and intracellular RONS levels. P values < 0.05 were considered statistically significant.
